# F1099L-CFTR (c.3297C>G) has Impaired Channel Function and Associates with Mild Disease Phenotypes in Two Pediatric Patients

**DOI:** 10.3390/life11020131

**Published:** 2021-02-08

**Authors:** Xiaoying Zhang, Jaspal S. Hothi, Yanhui H. Zhang, Aixia Ren, Michael J. Rock, Saumini Srinivasan, Dennis C. Stokes, Anjaparavanda P. Naren, Weiqiang Zhang

**Affiliations:** 1 Department of Pediatrics, College of Medicine, University of Tennessee Health Science Center, Memphis, TN 38103, USA; xiaoyingzhang@imm.ac.cn (X.Z.); JHothi2@dmc.org (J.S.H.); aixia.ren@stjude.org (A.R.); ssriniv2@uthsc.edu (S.S.); dennis.stokes@vumc.org (D.C.S.); 2 University of Tennessee Cystic Fibrosis Care and Research Center at Le Bonheur Children’s Hospital-Methodist University Hospital, Memphis, TN 38103, USA; 3 Department of Bioscience Research, College of Dentistry, University of Tennessee Health Science Center, Memphis, TN 38163, USA; yzhang36@uthsc.edu; 4 Department of Pediatrics, University of Wisconsin School of Medicine and Public Health, Madison, WI 53792, USA; mjrock@wisc.edu; 5 Department of Pediatrics, Cincinnati Children’s Hospital Medical Center, 3333 Burnet Avenue, R-4041, Cincinnati, OH 45229, USA; anaren@cchmc.org; 6 Department of Physiology, College of Medicine, University of Tennessee Health Science Center, Memphis, TN 38163, USA; 7 Children’s Foundation Research Institute, Le Bonheur Children’s Hospital, Memphis, TN 38103, USA

**Keywords:** protein channel, cystic fibrosis (CF), CFTR, F1099L-CFTR, CFTR modulators, VX-809 (lumacaftor), personalized medicine

## Abstract

(1) Background: many rare *cystic fibrosis* *transmembrane conductance regulator* (CFTR) mutations remain poorly characterized with regard to functional consequences of the mutation. We present the clinical features of two pediatric cystic fibrosis (CF) subjects who are heterozygous for F1099L (c.3297C>G), one with G551D (a class III mutation) and one with 3849 + 10kbC->T (a class V mutation). We also identified the molecular defect(s) that are associated with F1099L mutation to correlate with the clinical features that we observed; (2) Methods: clinical findings and history were extracted from the electronic medical record and de-identified. F1099L-CFTR protein expression level and maturation status, channel function, and the effects of CFTR modulation on these characteristics were investigated using western blotting and iodide efflux assay; (3) Results: these two subjects have mild CF phenotypes when F1099L is combined with two known disease-causing mutations. F1099L-CFTR has a moderate defect in processing and maturation, causing fewer CFTR channels at the cell surface and, therefore, impaired channel activities. These defects could be effectively corrected using VX-809 (lumacaftor); and, (4) Conclusions: our biochemical data correlate with the disease manifestations and suggest that F1099L is potentially a CF-causing mutation. The study expands our knowledge of rare CFTR mutations and may help in developing effective therapies for subjects with F1099L mutation.

## 1. Introduction

Cystic fibrosis (CF) is a life-shortening disease that is caused by mutations in the *CF transmembrane conductance regulator* (*CFTR*) gene that lead to the loss or dysfunction of CFTR channel activity [1,2]. Clinically, CF can affect multiple organs, including the upper airway, lungs, pancreas, sweat glands, intestine, liver, and vas deferens. The leading cause of morbidity and mortality for CF patients is chronic lung disease [3,4]. The incidence of CF and the frequency of specific mutations vary among ethnic populations [5,6].

CFTR is a cAMP/cGMP-regulated chloride (Cl^−^) and bicarbonate (HCO_3_^−^) channel that is primarily expressed at the apical (luminal) membrane of epithelial cells lining the airway, gut, and exocrine glands, where it regulates transepithelial fluid secretion and homeostasis [7,8,9]. CFTR is a member of the ATP-binding cassette transporter superfamily, and it consists of 1480 amino acids. CFTR is composed of two membrane-spanning domains (MSD1 and MSD2), two nucleotide binding domains (NBD1 and NBD2), and a regulatory domain (R) [7]. The CFTR channel can be activated through the phosphorylation of its R domain by various protein kinases (e.g., PKA) and binding and hydrolysis of ATP at its NBDs. CFTR channel activity is determined by the number, open probability, and conductance of channels at cell surface [10]. Mutations in the *CFTR* gene can alter one or more of these parameters, which causes the impairment or loss of the channel activity. Currently, 2103 CFTR mutations have been identified [11], which were traditionally categorized into six groups based on the nature of defect(s) [12,13]. Because some mutations have multiple defects, an expanded classification method was also proposed [14]. F508del is the most prevalent CFTR mutation; approximately 80–85% of CF patients carry it on at least one allele worldwide [15,16]. The classification of CFTR mutations helps to define strategies to restore CFTR channel function that is based on mutation-specific defect(s). The U.S. Food and Drug Administration has approved several CFTR modulators for CF therapy, including Trikafta™, Kalydeco^®^, Orkambi^®^, and Symdeko^®^ (Vertex Pharmaceuticals Inc., Boston, MA, USA) [17].

Among these 2103 known CFTR mutations, only a relative few have been studied in detail at both the molecular and phenotypic levels. Therefore, it is critical to study the molecular and clinical characteristics of rare CFTR mutations, particularly of those seen in minority populations, to identify the defect(s) and help develop effective therapies. In this paper, we present two clinical cases of pediatric CF subjects who have a missense mutation F1099L (c.3297C>G). We also characterized the mutation at the molecular level in order to identify the nature of defect(s).

## 2. Materials and Methods

### 2.1. Clinical Features

These two subjects received standard care at the University of Tennessee Cystic Fibrosis Research and Care Center at LeBonheur Children’s Hospital (Memphis, TN, USA). Their medical records and CF Foundation Registry data were retrospectively analyzed after Institutional Review Board approval (UTHSC 13-02779-XM). The clinical information was de-identified.

### 2.2. Antibodies and Reagents

Antibodies: anti-CFTR (clone MM13-4, EMD Millipore Corporation, CA, USA), anti-CFTR (ACL-006, Alomone labs, Jerusalem, Israel), anti-β-actin (Sigma, MO, USA), goat anti-mouse IgG secondary antibody, HRP (Pierce Biotechnology, IL, USA), and goat anti-rabbit IgG Alexa Fluor 488 (Thermo Fisher Scientific, MA, USA). VX-809 (Selleckchem, TX, USA). Other reagents that were used in this study were purchased from Sigma or Fisher Scientific (PA, USA).

### 2.3. Generation of F1099L-CFTR Mutation (cDNA Name: c.3297C>G) 

QuikChange™ Site-Directed Mutagenesis Kit (Stratagene, CA, USA) was used to generate c.3297C>G point mutation on a pcDNA3.1-wild type (WT)-CFTR background. The primers used were:

Forward: 5′CCTGTCAACACTGCGCTGGTTGCAAATGAGAATAGAAATG3′

(TTC > TTG, amino acid F > amino acid L)

Reverse: 5′CATTTCTATTCTCATTTGCAACCAGCGCAGTGTTGACAGG3′

We confirmed all of the sequences at the Molecular Resource Center at The University of Tennessee Health Science Center. 

### 2.4. Cell Culture and Plasmids Transfection

WT and CFTR mutant were expressed in HEK-293 cells (ATCC^®^ CRL-1573™, Manassas, VA, USA). The cells were grown in DMEM/F12(1:1) medium (Invitrogen, NY, USA) containing 1% penicillin-streptomycin (Invitrogen) and 10% fetal bovine serum (Invitrogen) supplements, and then incubated at 37 °C with 5% CO_2_, unless otherwise stated. The cells were transfected using Lipofectamine 2000 (Invitrogen) following the manufacturer’s instruction. At 48 h post-transfection, the cells were used for Western blotting or iodide efflux assay.

### 2.5. Western Blotting

The cells were lysed in a lysis buffer [1× PBS, containing 0.2% Triton-X-100 and protease inhibitors (cOmplete™, Roche, IN, USA)] for 30 min. at 4 °C, and centrifuged at 12,000 rpm for 15 min. at 4 °C. The total protein levels of supernatants were measured by using Bradford protein assay. The supernatants were mixed with 4× sample buffer, denatured, subjected to SDS-PAGE on 5–14% Gel (Bio-Rad, CA, USA), and then transferred to PVDF membranes (Pierce Biotechnology). The membranes were blocked with blocking buffer (5% milk in 0.1% PBS-T) and then probed with respective antibody against CFTR (MM13-4, 1:1,000 dilution) or β-actin (1:5000 dilution). The protein bands were visualized while using ECL™ Western blot detection reagents (GE Healthcare, Buckinghamshire, UK) and quantified using ImageJ software (US National Institutes of Health). The Supplemental Materials provided the whole blots and densitometry readings and analysis of the bands of interest (Figures S1–S3). 

### 2.6. Real-Time PCR to Measure CFTR mRNA Levels

The total RNA was isolated from HEK-293 cells transfected with WT- or F1099L-CFTR using Purelink RNA Mini Kit (Thermo Fisher Scientific). One microgram (1 μg) of total RNA was converted to cDNA using SuperScript III Reverse Transcriptase (Invitrogen). Real-time PCR was performed using LightCycler 480 (Roche, Indianapolis IN, USA). The primers for human CFTR were: 

Forward: 5′TTGGATCCAGTAACATACC3′

Reverse: 5′TCAGCAGTTTCTGGATGGAATCG3′

The primers for human GAPDH were: 

Forward: 5′TGATGACATCAAGAAGGTGG3′

Reverse: 5′TCGTTGTCATACCAGGAAATG3′ 

The parameters for PCR thermocycling were: 95 °C for 5 min., 95 °C for 10 s (40 cycles), and 60 °C for 30 s. All of the samples were run in triplicate. The levels of CTFR mRNAs were normalized to GAPDH.

### 2.7. Iodide (I^−^) Efflux Assay

Cells were grown on poly-lysine-coated 60-cm culture dishes and then transfected with WT-CFTR, F1099L-CFTR, or empty vector using Lipofectamine 2000. At 48 h post transfection, the culture medium was removed, and the cells were loaded for 60 min. at room temperature with an I^-^-containing loading buffer (136 mM NaI, 137 mM NaCl, 4.5 mM KH_2_PO_4_, 1 mM CaCl_2_, 1 mM MgCl_2_, 10 mM glucose, and 5 mM HEPES, pH 7.2). After removing the loading buffer, the cells were washed five times with an efflux buffer (136 mM NaNO_3_ replacing 136 mM NaI in the loading buffer) to remove the extracellular NaI. One milliliter (1 mL) of the efflux buffer was then added to each culture dish and the samples were collected after 1 min. The procedure was repeated another three times (note: these four samples were used to establish a stable baseline I^−^ efflux for each culture dish). Subsequently, a cocktail of PKA activating agonists (final concentrations: 10 µM forskolin, 200 µM CPT-cAMP, and 100 µM IBMX) were added to the efflux buffer to activate the CFTR channel and another six samples were collected from each dish to measure the CFTR-mediated I^-^ efflux. The I^−^ concentrations in the collected samples were measured using a combination iodide electrode (9653BNWP, Orion Research Inc., Chelmsford, MA, USA). The I^-^ efflux rates were reported as nano-moles (nmol)/min. Upon the completion of sample collection, the cells were lysed and the total protein concentrations were measured using Bradford protein assay. The maximal I^-^ efflux rates of samples were normalized to their total protein concentrations of cell lysates. 

### 2.8. Immunofluorescence Labeling and Imaging

HEK-293 cells were grown on poly-lysine-coated microscope cover glasses and then transfected with F1009L-CFTR, WT-CFTR, or vector plasmids. After 24 h, VX-809 (5 µM) or DMSO was added and cells were cultured for another 24 h. The cells were washed twice with PBS, fixed with 4% formaldehyde for 10 min., permeabilized with 0.1% Triton-X-100 for 5 min., and then blocked with goat serum (1% in PBS) for 30 min. at room temperature. The cells were incubated with a CFTR polyclonal antibody (ACL-006: 1:50) at 4 °C overnight, washed five times with PBS, and incubated with the secondary anti-rabbit Alexa Fluor 488 (1:2000) for 1 h. The cover glasses were mounted with DAPI Fluoromount-G (SouthernBiotech, AL, USA). Fluorescence images were taken on a Zeiss 710 LSM microscope while using a 60× objective.

### 2.9. Statistical Analysis

The data were reported as Mean ± S.E.M (standard error of the mean). Student’s t-test was used for statistical analysis and *p* values < 0.05 were considered to be significant.

## 3. Results

### 3.1. Clinical Features

We have two subjects identified with the F1099L mutation; both are pancreatic sufficient and with mild clinical phenotypes.

**Subject #1**: an African American infant was diagnosed at 23 months old with failure to thrive, hypochloremic metabolic alkalosis, and elevated sweat chloride levels (99, 116 mmol/L). Newborn screening was falsely negative (immunoreactive trypsinogen (IRT) cut-off at the time was >100 ng/ml for a second IRT testing). Repeat sweat chloride tests at age three and seven years showed values of 84 and 104 mmol/L. Genotyping revealed G551D on one chromosome and F1099L on the other. Stool elastase testing at 23 months and four ½ years old showed pancreatic sufficiency (>500 µg PE/g). Respiratory cultures have been positive for methicillin-resistant Staphylococcus aureus, with no cultures positive for Pseudomonas aeruginosa. The subject had low vitamin D levels. Chest radiograph was initially normal and has remained normal but with some increased markings. At six years of age, the subject was started on Kalydeco^®^ for treatment of G551D mutation. Repeat sweat chloride testing at nine years old showed levels of 72.5 mmol/L and 79.5 mmol/L (~19% reduction). Previously, low vitamin D levels are now normal with the most recent value of 46.2 ng/mL. Growth and lung function have remained normal. 

**Subject #2:** a mixed race infant was diagnosed by positive newborn screening at one month of age in Wisconsin, USA. The subject had normal or intermediate sweat chloride levels at four months (24, 32 mmol/L) and 10 months old (43 and 41 mmol/L). Genotyping revealed that the subject is heterozygous for 3849+10kbC->T and F1099L. Stool elastase testing at six months of age was normal (>500 µg PE/g) and repeats at three ½ years (236 µg PE/g) and nine years (>500 µg PE/g) demonstrated pancreatic sufficiency. Respiratory cultures have been primarily positive for methicillin-resistant Staphylococcus aureus, with one non-mucoid Pseudomonas aeruginosa isolate at six months of age, and recently positive for methicillin-sensitive Staphylococcus aureus and Streptococcus pyogenes, group A. Pulmonary function tests, chest radiograph, and growth have remained normal. 

### 3.2. F1099L-CFTR Has a Defect in Protein Maturation and Exhibits Impaired Channel Function Compared to WT-CFRT

**Figure 1:**
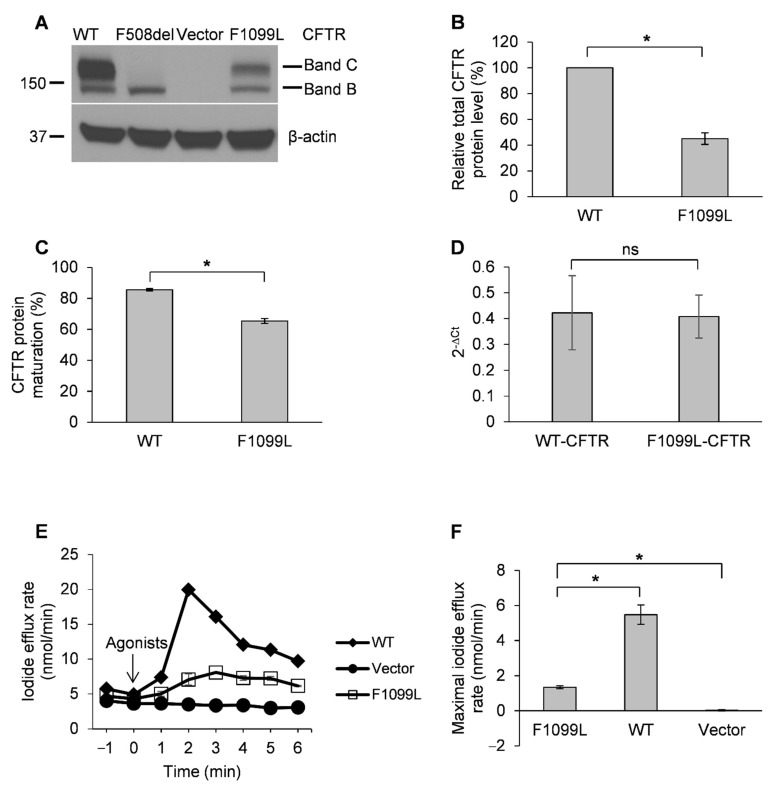
F1099L-*cystic fibrosis transmembrane conductance regulator* (CFTR) has a defect in protein maturation and exhibits impairment in chloride channel function. (**A**) A representative blot showing the expression level and maturation status of F1099L-, wild type (WT)-, or F508del-CFTR, or vector transiently expressed in HEK-293 cells. Band C and B denote a mature form and an immature form of CFTR, respectively. (**B**) The total CFTR protein expression level (Band C + Band B) of F1099L-CFTR was 45% of WT-CFTR. The data were quantified from blots as represented in (**A**) using ImageJ software. The densities of the bands were normalized to their loading control, β-actin, respectively. * *p* < 0.05, *n* = 3. (**C**) F1099L-CFTR has a defect in protein maturation, with a mean maturation efficiency of 65%. The maturation efficiency was defined as a ratio of the quantity of mature form CFTR (the density of Band C) to the quantity of total CFTR protein (the density of Band C plus Band B). * *p* < 0.05, *n* = 3. (**D**) CFTR mRNA levels were comparable in HEK-293 cells transfected with WT- or F1099L-CFTR plasmid. ns: not significant. *n* = 9. (**E**) Representative iodide efflux traces of F1099L-CFTR, WT-CFTR, or vector transiently expressed in HEK-293 cells. Upon stimulation, F1099L-CFTR exhibited an impairment in channel function. PKA activating agonists (final concentrations): 10 μM forskolin, 100 μM IBMX, and 200 μM cpt-cAMP. (**F**)The mean maximal iodide efflux rate of F1099L-CFTR was 23% of WT-CFTR. The data were quantified from experiments as represented in (**E**) and normalized to the total protein concentrations of assay samples, respectively. * *p* < 0.05, *n* = 6.

The cDNA of F1099L mutation, c.3297C>G, was generated on a pcDNA3.1-WT-CFTR background using site-directed mutagenesis. The plasmids of WT-CFTR, F1099L-CFTR, F508del-CFTR, or empty vector were transfected into HEK-293 cells and Western blotting was used in order to test the expression level and maturation status of CFTR proteins. WT-CFTR was expressed predominantly as a mature form (Band C) (Figure 1A) with a maturation efficiency of 86%, as shown in Figure 1 (Figure 1C). F508del-CFTR was expressed as an immature form (Band B) and no CFTR protein could be detected in the vector-transfected cells (Figure 1A). F1099L-CFTR was expressed in both mature and immature forms (Figure 1A), with a maturation efficiency of 65% (Figure 1C). The total F1099L-CFTR protein expression level was 45% of WT-CFTR (Figure 1B). We next measured mRNA levels of F1099L-CFTR and WT-CFTR using real-time PCR. We found that their levels were comparable (Figure 1D). Our data suggest that F1099L-CFTR has a defect in intracellular processing and trafficking, which leads to impaired protein maturation and, hence, fewer mature protein at the plasma membrane.

The channel function of F1099L-CFTR was measured in HEK-293 cells using iodide efflux assay. HEK-293 cells that were transfected with WT-CFTR or vector were used as controls. WT-CFTR exhibited a typical CFTR-mediated iodide efflux profile, whereas the vector-transfected cells did not show any measurable iodide efflux, as shown in Figure 1E. F1099L-CFTR elicited a response; however, the magnitude was markedly smaller when compared with WT-CFTR. The normalized maximal iodide efflux rate of F1099L-CFTR was 23% of that of WT-CFTR (Figure 1F). Our data demonstrate that, as compared to WT-CFTR, the channel activity of F1099L-CFTR is impaired, which correlates with our finding that this mutant CFTR protein has a defect in intracellular trafficking and maturation.

### 3.3. F1099L-CFTR Is a Temperature-Sensitive Mutation and Can Be Effectively Rescued by Using VX-809

**Figure 2:**
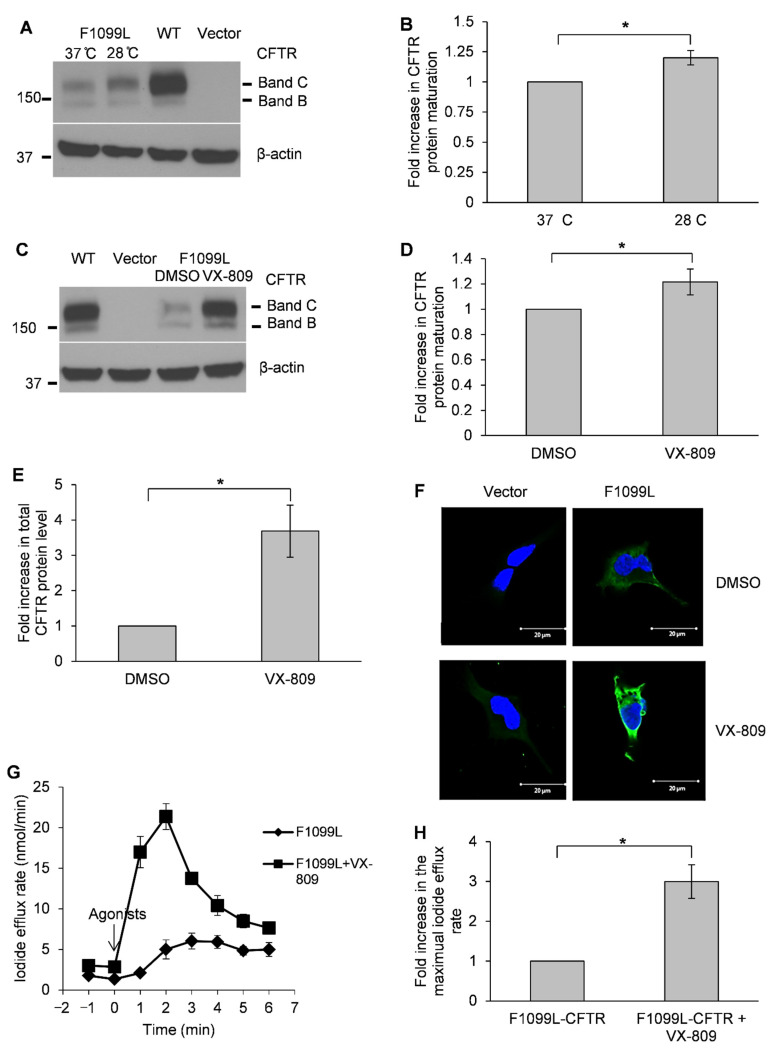
F1099L-CFTR is a temperature-sensitive mutation and can be effectively rescued by using VX-809. (**A**) F1099L-CFTR is a temperature-sensitive mutation. Shown here is a representative blot of F1099L-CFTR in HEK-293 cells cultured at physiological temperature (37 °C) or at a low temperature (28 °C). WT-CFTR- and vector-transfected HEK-293 cells were cultured at 37 °C and used as controls. (**B**) Low temperature (28 °C) promoted the maturation efficiency of F1099L-CFTR (1.2-fold increase vs. 37 °C)**.** The data were quantified from blots as represented in (**A**) using ImageJ software. * *p* < 0.05, *n* = 3. (**C**) F1099L-CFTR was effectively rescued by using VX-809. Shown here is a representative blot of F1099L-CFTR in HEK-293 cells that were treated with VX-809 (5 μM) or DMSO. WT-CFTR- and vector-transfected HEK-293 cells were used as controls. (**D**) VX-809 increased the maturation efficiency of F1099L-CFTR (1.2-fold vs. DMSO). The data were quantified from blots as represented in (**C**) using ImageJ software. * *p* < 0.05, *n* = 3. (**E**) VX-809 increased the total CFTR protein level of F1099L-CFTR (3.7-fold vs. DMSO). The data were quantified from blots as represented in (**C**) using ImageJ software and normalized to β-actin, respectively. * *p* < 0.05, *n* = 3. (**F**) VX-809 increased the total CFTR protein level of F1099L mutation and promoted its maturation as evidenced by immunofluorescence imaging data. F1099L-CFTR- and vector-transfected HEK-293 cells were treated with VX-809 (5 μM) or DMSO and then subjected to immunofluorescence labeling and imaging. (**G**) VX-809 augmented the channel function of F1099L-CFTR. Shown here are representative iodide efflux traces of F1099L-CFTR in HEK-293 cells treated with VX-809 (5 μM) or DMSO. *n* = 3. (**H**) The mean maximal iodide efflux rate of F1099L-CFTR increased three-fold with VX-809 treatment compared to DMSO-treated controls. The data were quantified from experiments as represented in (F) and normalized to the total protein concentrations of assay samples, respectively. * *p* < 0.05, *n* = 9.

Because F1099L-CFTR exhibited a defect in protein maturation, which shares a characteristic that is associated with the Class II CFTR mutations, we next investigated whether the maturation defect of F1099L-CFTR can be corrected using a CFTR corrector VX-809 or exposure to low temperature (28 °C). HEK-293 cells transfected with F1099L-CFTR plasmid were cultured at 37 °C or 28 °C to test the temperature effect. Cells that were transfected with WT-CFTR or vector were used as controls. We found that, as compared to cultures at 37 °C, exposure to low temperature promoted the maturation of F1099L-CFTR (1.2-fold) and increased the total CFTR protein level (1.7-fold) (Figure 2A,B). To test VX-809 effect, the HEK-293 cells were transfected with F1099L-CFTR plasmid and then treated with VX-809 (5 μM) or DMSO (negative control) and cultured at 37 °C. We found that VX-809 markedly increased the total F1099L-CFTR protein level (3.7-fold) and significantly promoted its maturation (1.2-fold) (Figure 2C–E). Immunofluorescence imaging data also showed that VX-809 increased the total level and maturation of F1099L-CFTR (Figure 2F and Figure S4).

We next tested the VX-809 effect on the F1099L-CFTR channel function in HEK-293 cells using iodide efflux assay. We found that VX-809 augmented F1099L-CFTR channel function, with a three-fold increase in the maximal iodide efflux rate when compared to DMSO control (Figure 2G,H).

## 4. Discussion

Newborn screening programs and full-length CFTR sequencing have facilitated the identification of rare or unique CFTR mutations, especially in minority populations where classical CF phenotypes are uncommon [5,6]. Many of these rare mutations have not been studied at the molecular level, and the nature of the defects has not been identified. The lack of this knowledge hinders the development of effective and personalized therapies in minority populations.

We identified a missense mutation F1099L in two pediatric CF patients. We characterized this mutation at the molecular level while using a heterologous cell expression model, and found that (1) F1099L-CFTR has a defect in protein maturation. When compared to WT-CFTR, the total protein level and maturation efficiency of F1099L mutant were significantly decreased; (2) F1099L-CFTR has an impaired channel function (23% of WT-CFTR); (3) F1099L-CFTR is a temperature-sensitive mutant; lowering the temperature promoted the protein maturation; and, (4) F1099L-CFTR responded very well to VX-809 treatment. The total CFTR protein level and the channel function were markedly increased (3.7- and three-fold, respectively) with the use of VX-809, to levels almost comparable to WT-CFTR.

Our molecular characterization of F1099-CFTR was conducted in HEK-293 cells. Although HEK-293 is one of the most commonly used cell lines for studying CFTR protein expression and channel function, caution should be exercised when trying to associate these data with clinical findings. For the purpose of developing personalized medicine for these two or other patients with a F1099L mutation, a better approach is to harvest the rectal or trachea/bronchial biopsies and test their responses to CFTR modulators in a Ussing chamber, or to convert these biopsies into organoids and use the forskolin-induced swelling assay to test their responses [18]. The CFTR modulators to be tested should include the active components of Trikafta™: VX-661 (Tezacaftor), VX-445 (Elexacaftor), and VX-770. Currently, there are seven patients with F1099L in the CFTR2 database. F1099L was listed as a mutation with varying clinical consequences [19]. By using extensive sequencing of *CFTR* gene, McGinniss and colleagues identified F1099L in a pediatric patient who has the genotype of F508del/F1099L and showed positive newborn IRT and borderline sweat chloride levels (48, 52 mmol/L) [20]. Degrugillier et al. reported a clinical case of a patient with F508del and F1099L who had severe chronic rhinosinusitis. The authors found that F1099L-CFTR matured less fully than WT-CFTR and that VX-809 alone or in combination with VX-770 promoted the maturation of F1099L-CFTR, to a level that is comparable to WT-CFTR. They did not find significant difference in the correction efficiency between VX-809 alone and a combination of VX-809 and VX-770 [21]. Recently, Dr. Cutting’s group assessed the function of 48 *CFTR* missense variants in CF bronchial epithelial cells (CFBE41o-). They found that F1099L-CFTR reduced the chloride conductance to 15.1 ± 6.4% of WT-CFTR [22]. Our data are consistent with these findings. The independent and corroborative results from different research groups consolidate our understanding of F1099L mutation and help to develop effective therapies for patients with this mutation.

It has been reported that, in addition to F508del, VX-809 also worked on other CFTR mutations, although with varying efficacies [23,24]. In a previous study, we found that VX-809 could partially rescue the expression and function of a G1208D mutation [24]. VX-809 was more efficacious for rescuing F1099L than for G1208D. F1099L-CFTR is located in the 4th cytosolic loop (CL4) between the transmembrane domain TM10 and TM11 based on the Swiss-Prot numbering system or the original Science paper number [25]. A cluster of mutations have been found in CL4 that cause misfolding of the CFTR protein; examples included R1070W, R1070Q, F1074L, A1067T, and R1066H [16,26,27]. CL4 has been shown to interact with NBD1, and this interface is important for CFTR folding [28]. Studies on the three-dimensional structures of WT, F508del, and these other responding mutations could help to unveil the mechanisms of action of VX-809 and other newer correctors (e.g., Tezacaftor and Elexacaftor), and develop more effective CFTR correctors. 

It is known that the pulmonary disease phenotypes do not correlate well with CFTR genotypes, and that other genetic (e.g., modifier genes) and environment factors (e.g., socioeconomic status) affect the disease severity [29,30,31,32]. Our finding that F1099L-CFTR has a residual CFTR function (23% of normal CFTR) seems to correlate with the mild disease phenotypes that were shown in these two subjects, with both having pancreatic sufficiency and normal chest radiograph. This finding also correlates with the sweat chloride levels in these two subjects: when paired with G551D in patient #1, the sweat chloride levels were high (84–116 mmol/L); when paired with 3849+10kbC->T (which usually associates with mild disease presentation) in patient #2, the sweat chloride levels were low in the range of 24–43 mmol/L.

Although our patients currently showed mild CF disease phenotypes, we cannot, as of yet, predict the effect of F1099L mutation on disease progression. It is known that patients with residual CFTR channel activity can still develop bronchiectasis with chronic sino-pulmonary infections and other CF-related diseases when they get older [12]. Given that F1099L was very responsive to VX-809 correction, we speculate that VX-809 and similar CFTR modulators (e.g., Tezacaftor and Elexacaftor) could be beneficial for patients with this mutation. For subject #1, who has G551D and F1099L mutations, it might be interesting to test whether Orkambi^®^, Symdeko^®^, or Trikafta™ might be a better option for therapy than Kalydeco^®^ alone by using a personalized medicine approach if his clinical status worsens on ivacaftor alone.

## 5. Conclusions

We report the clinical features of two pediatric CF patients having a F1099L mutation. Subject #1 is heterozygous for F1099L and G551D. Subject #2 is heterozygous for F1099L and 3849+10kbC->T. Both patients showed mild disease phenotypes. We characterized F1099L mutation using a heterologous expression system and found the mutation resulted in a defect in protein processing and maturation, causing fewer functional proteins at the cell surface and therefore impaired channel activities. Our data suggest that F1099L mutation is potentially a disease-causing mutation and that its maturation defect can be effectively rescued by using a CFTR corrector VX-809. The independent and corroborative findings from our study and others groups expand our knowledge of F1099L-CFTR mutation and could facilitate the development of effective therapies for patients with this mutation.

## Data Availability

Data is contained within the article or supplemental material.

## Supplementary Materials

The following are available online at https://www.mdpi.com/2075-1729/11/2/131/s1, Figure S1: Original Western blot images of Figure 1A in the manuscript, Figure S2: Original Western blot images of Figure 2A in the manuscript, Figure S3: Original Western blot images of Figure 2C in the manuscript, Figure S4: VX-809 increased the total CFTR protein level of F1099L mutation and promoted its maturation as evidenced by immunofluorescence imaging data.
