# Supplementary material for: F1099L-CFTR (c.3297C>G) has Impaired Channel Function and Associates with Mild Disease Phenotypes in Two Pediatric Patients
Source: Life (Basel). 2021 Feb 8;11(2):131. doi: 10.3390/life11020131 (PMC7915716; doi:10.3390/life11020131)
Supplement: Supplementary file 1 [file life-11-00131-s001.pdf]

life

MDPI

Article

F1099L-CFTR (c.3297C>G) has Impaired Channel Function and Associates with Mild Disease Phenotypes in Two Pediatric Patients

Xiaoying Zhang [1], Jaspal S. Hothi [1,2], Yanhui H. Zhang [3], Aixia Ren [1], Michael J. Rock [4], Saumini Srinivasan [1,2], Dennis C. Stokes [1,2], Anjaparavanda P. Naren [5] and Weiqiang Zhang [1,2,6,7,*]

[1] Department of Pediatrics, College of Medicine, University of Tennessee Health Science Center, Memphis, TN 38103, USA; xiaoyingzhang@imm.ac.cn (X.Z.); JHothi2@dmc.org (J.S.H.); aixia.ren@stjude.org (A.R.); ssriniv2@uthsc.edu (S.S.); dennis.stokes@vumc.org (D.C.S.)
[2] University of Tennessee Cystic Fibrosis Care and Research Center at Le Bonheur Children's Hospital - Methodist University Hospital, Memphis, TN 38103, USA
[3] Department of Bioscience research, College of Dentistry, University of Tennessee Health Science Center, Memphis, TN 38163, USA; yzhang36@uthsc.edu
[4] Department of Pediatrics, University of Wisconsin School of Medicine and Public Health, Madison, WI 53792, USA; mjrock@wisc.edu
[5] Department of Pediatrics, Cincinnati Children's Hospital Medical Center, 3333 Burnet Avenue, R-4041, Cincinnati, OH 45229, USA; anaren@cchmc.org
[6] Department of Physiology, College of Medicine, University of Tennessee Health Science Center, Memphis, TN 38163, USA
[7] Children's Foundation Research Institute, Le Bonheur Children's Hospital, Memphis, TN 38103, USA
* Correspondence: wzhang16@uthsc.edu; Tel.: +1-(901)-287-5367

Citation: Zhang, X.; Hothi, J.S.; Zhang, Y.H.; Ren, A.; Rock, M.J.; Srinivasan, S.; Stokes, D.C.; Naren, A.P.; Zhang, W. F1099L-CFTR (c.3297C>G) has Impaired Channel Function and Associates with Mild Disease Phenotypes in Two Pediatric Patients. *Life* **2021**, *11*, 131. https://doi.org/10.3390/life11020131

Received: 24 December 2020
Accepted: 05 February 2021
Published: date

Publisher's Note: MDPI stays neutral with regard to jurisdictional claims in published maps and institutional affiliations.

Copyright: © 2021 by the authors. Submitted for possible open access publication under the terms and conditions of the Creative Commons Attribution (CC BY) license (http://creativecommons.org/licenses/by/4.0/).

Supplementary Materials

Note:

The densities of Western blots bands were quantified using ImageJ software (U.S. National Institutes of Health). We followed the method of 'Analyzing gels and western blots with ImageJ' (https://lukemiller.org/index.php/2010/11/analyzing-gels-and-western-blots-with-image-j/).

The CFTR maturation efficiency was defined as a ratio of the mature form CFTR (density of Band C) to the total CFTR protein (density of Band B plus Band C).

The CFTR total protein level was defined as the density of Band B plus Band C. Prior to comparisons, the CFTR bands densities were first normalized to their respective β-actin levels (bands densities).

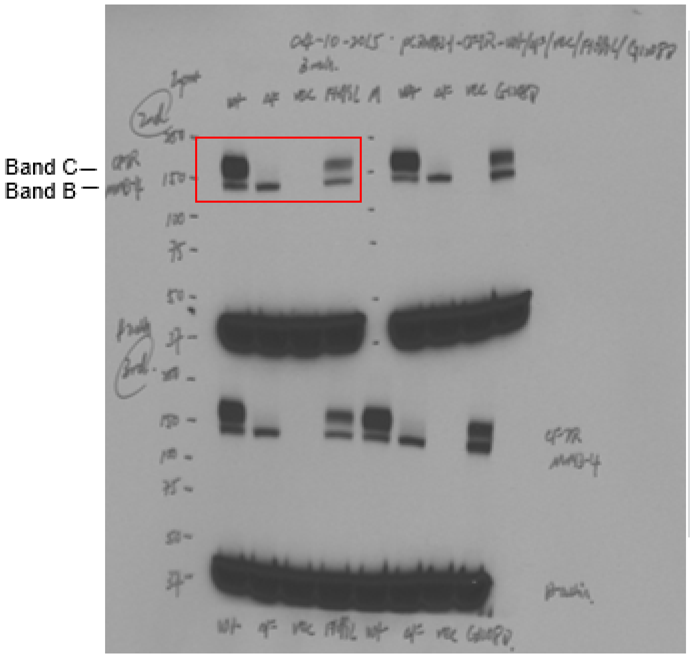

(a)

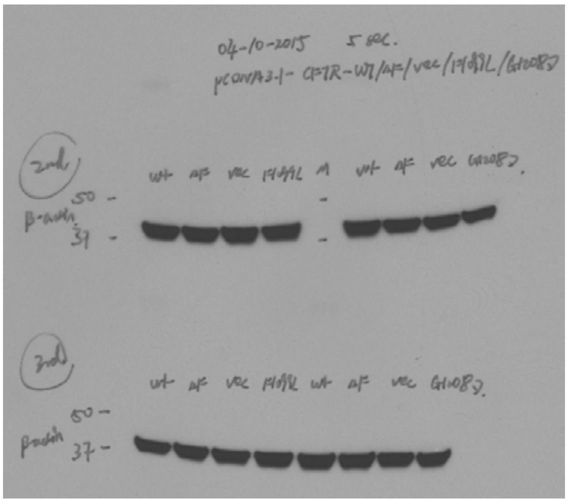

(b)

				**2nd Exp_04-10-2015**									
	**1. Band c/ total (band b + c)**												
				Raw data (Area)									
					total (Area)		band c/total		band c /total protein (%)				
		**WT band C**		55940.37			0.863074		86.3074				
		WT band B		8874.898	64815.27								
		**F1099L band C**		28736.15									
		F1099L band B		16317.58	45053.74		0.63782		63.78195				
	**2. Total protein**			Using the 'straight line' tool to cover the whole area.									
				Raw data		Ajusted (protein/actin)							
	WT			127205.5		1.361107							
	WT_actin			93457.34									
	F1099L			67753.56									
	F1099L_actin			100471.3		0.674358							
	F1099L/WT (normalized to actin)					0.495448							

(c)

Figure S1. Original Western blot images of Figure 1A in the manuscript. **(a)** (In the red Text Box) from left to right: WT-CFTR, F508del-CFTR, Vector, and F1099L-CFTR. Exposure time: 3 min. The Western blotting experiments were repeated 3 times. Shown in this blot were the 2nd and the 3rd experiments. The blot from the 2nd experiment (in the red Text Box) was used as the representative blot in the manuscript (Figure 1A). **(b)** Original image of β-actin exposed for 5 s. The blot in the red Text Box was used in Figure 1A. **(c)** Densitometry readings of WT-CFTR and F1099L-CFTR bands using ImageJ software. Maturation efficiency (Band c/total (band b+c): WT-CFTR (86.3%), F1099L-CFTR (63.8%). Total protein (F1099L was 49.5% of WT-CFTR).

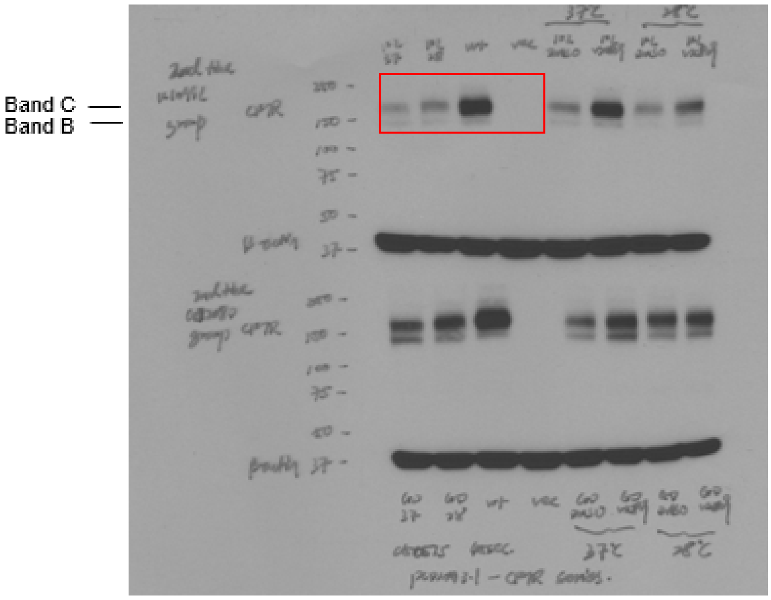

(a)

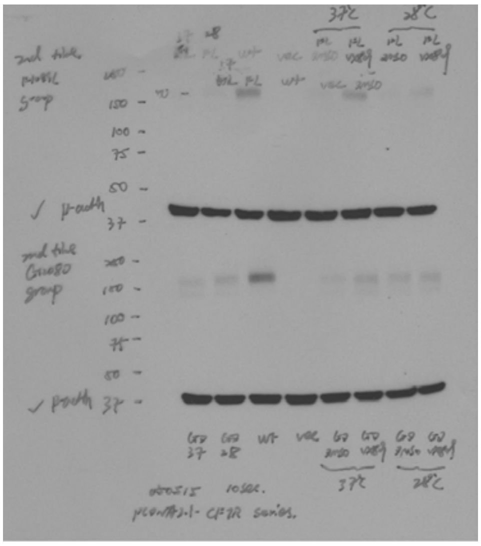

(b)

	**1. Band c/ total (band b + c)**		Note: using method option 2				
		Raw data (Area)					
			**total (Area)**	**band c/total**	**band c /total protein (%)**	**fold change**	**% of WT**
	**Temp sensitivity**						
	**37 C_Band C**	12782.47					
	**37 C_Band B**	2618.175	15400.65	0.829996	82.99958	1	83.62095
28 C	**Band C**	23597.24					
	**Band B**	1599.447	25196.69	0.936522	93.65215	1.128345	94.35328
WT_37	**Band C**	61725.28					
	**Band B**	462.104	62187.39	0.992569	**99.25692**		**100**

(c)

Figure S2. Original Western blot images of Figure 2A in the manuscript. **(a)** (In the red Text Box) from left to right: F1099L-CFTR (37 °C), F1099L-CFTR (28 °C), WT-CFTR, and Vector. The Western blotting experiments were repeated 3 times. Shown in this blot was the 2nd experiment. The bands in the red Text Box were used as the representative blot in the manuscript (Figure 2A). Exposure time: 45 s. **(b)** Original image of β-actin exposed for 10 sec. The blot in the red Text Box was used in Figure 2A. **(c)** Densitometry readings of F1099L-CFTR bands at different temperature using ImageJ software. Fold change in maturation efficiency (1.13-fold at 28 °C compared to at 37 °C).

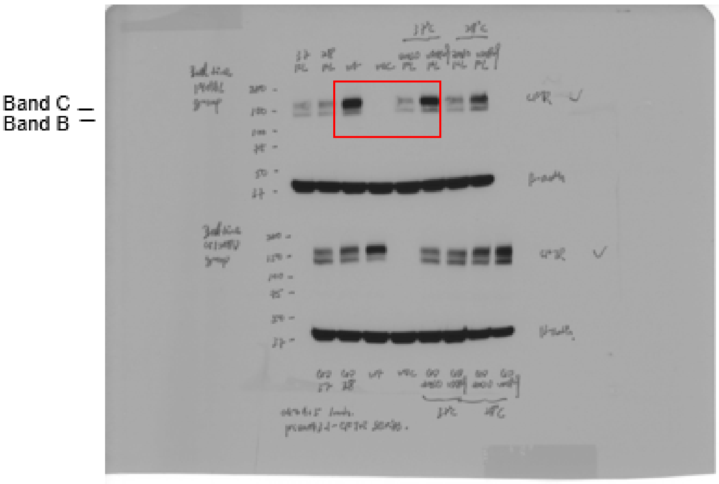

(a)

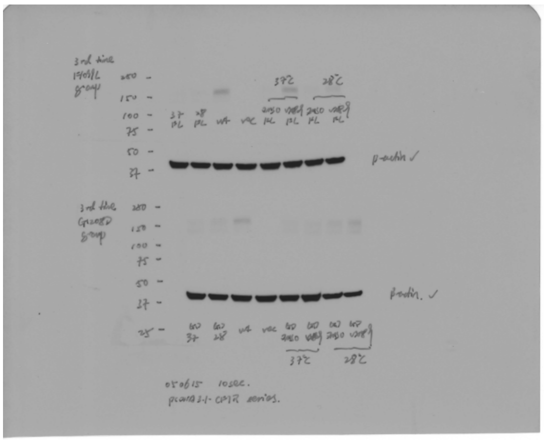

(b)

Exp.3_05-06-2015

1. Band c/ total (band b + c) Note: using method option 2

		Raw data (Area)	total (Area)	band c/total	band c /total protein (%)		fold change	% of WT
	**Temp sensitivity**							
	**37 C_Band C**	7894.702						
	**37 C_Band B**	3539.317	11434.02	0.690457	69.04573	(control)	1	72.72374
28 C	**Band C**	12444.77						
	**Band B**	3145.832	15590.6	0.798222	79.82225		1.156078	84.07431
WT_37	**Band C**	52993.43						
	**Band B**	2822.915	55816.34	0.949425	94.94249			**100**
	**VX-809 effect**							
DMSO	**37 C_Band C**	10107.09						
	**37 C_Band B**	3142.539	13249.63	0.762821	76.28206		1	80.34554
VX-809	**37 C_Band C**	50233.39						
	**37 C_Band B**	4683.472	54916.86	0.914717	91.4717		1.199125	96.34432
DMSO	**28 C_Band C**	14564.91						
	**28 C_Band B**	2905.953	17470.86	0.833669	83.36686		1.092876	87.80774
VX-809	**28 C_Band C**	30135.22						
	**28 C_Band B**	6317.007	36452.23	0.826705	82.67045		1.083747	87.07424

2. Total protein (Band B + Band C)

			Raw data	Ajusted (protein/actin)		Fold change	% of WT
	**Temp sensitivity**						
	**37 C_Band C**		**11434.02**				
	**37 C_Band B**	Actin	58588.06	0.19516	(control)	1	20.55954
28 C	**Band C**		15590.6				
	**Band B**	Actin	55389.87	0.28147		1.442257	29.65214
WT_37	**Band C**		55816.34				
	**Band B**	Actin	58801.04	0.949241			100
	**VX-809 effect**						
DMSO	**37 C_Band C**		13249.63				
	**37 C_Band B**	Actin	61557.23	0.215241	(control)	1	22.67505
VX-809	**37 C_Band C**		54916.86				
	**37 C_Band B**	Actin	60061.35	0.914346		4.248015	96.32393
DMSO	**28 C_Band C**		17470.86				
	**28 C_Band B**	Actin	56403.18	0.30975		1.439084	32.63129
VX-809	**28 C_Band C**		36452.23				
	**28 C_Band B**	Actin	52746.33	0.691086		3.210756	72.80404

(c)

Figure S3. Original Western blot images of Figure 2C in the manuscript. **(a)** (In red Text Box) from left to right: WT-CFTR, Vector, F1099L-CFTR (with DMSO), F099L-CFTR (VX-809). The Western blotting experiments were repeated 3 times. Shown in this blot was the 3rd experiment. The blot in the red Text Box was used as the representative blot in the manuscript (Figure 2C). Exposure time: 1 min. **(b)** Original image of β-actin exposed for 10 s. The blot in the red Text Box was used in Figure 2C. **(c)** Densitometry readings of F1099L-CFTR bands with VX-809 or DMSO treatment at different temperature using ImageJ software. VX-809 increased the maturation efficiency (1.2-fold) and total protein (4.2-fold) of F1099L-CFTR.

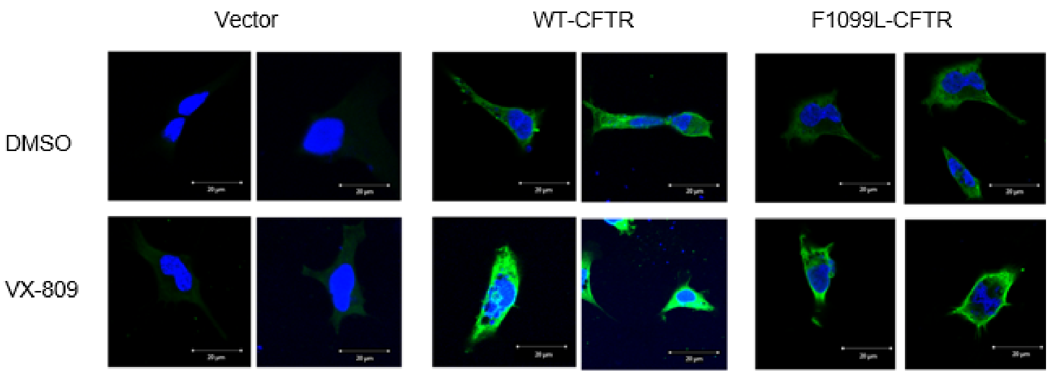

Figure S4. VX-809 increased the total CFTR protein level of F1099L mutation and promoted its maturation as evidenced by immunofluorescence imaging data. F1099L-CFTR-, WT-CFTR-, and vector-transfected HEK-293 cells were treated with VX-809 (5 μM) or DMSO and then subjected to immunofluorescence labeling and imaging.
